# A Neonatal Patient Diagnosed with Chromosome 18p 11.1 Microdeletion Syndrome Presented with Trisomy 18Like Phenotype

**DOI:** 10.1155/2023/2275582

**Published:** 2023-03-11

**Authors:** Deepa Banker, Bhavdeep Mungala, Zankhana Parekh, Shachi Ganatra, Vimal Maheshwari, Yashica Raj, Utsav Patel, Digant Patel, Kishan Chamar, Vasu Solanki

**Affiliations:** Department of Pediatrics, Smt. NHL Municipal Medical College, Ahmedabad, Gujarat, India

## Abstract

Microdeletion of the short arm of chromosome 18 is one of the most common chromosome deletion syndromes. Its estimated frequency is 1 in 50,000 live-born infants, with female prevalence over males. Around 150 cases have been described till now. The reported abnormalities include growth deficiency, hypotonia, microcephaly, dysmorphic facial features such as ptosis, epicanthal folds, hypertelorism and micrognathia, and relatively small hands and feet. Our patient was a full-term low birth weight (2150 gm) female newborn, showing cleft upper lip and palate (hard and soft palate), bilateral congenital Talipes Equinovarus with rocker bottom foot, microcephaly, atrial septal defect. She was initially conservatively managed with gavage feeding, then shifted into paladai feeding of expressed breast milk. A multidisciplinary approach was adopted due to various malformations and for the potential occurring complications. To our knowledge, this is the first case diagnosed during the neonatal period.

## 1. Introduction

An estimated 2,40,000 newborns die worldwide within 28 days of birth every year due to birth defects [1]. Microdeletion of the short arm of chromosome 18 is one of the most common chromosome deletion syndromes [2]. Its estimated frequency is 1 in 50,000 live-born infants, with female prevalence over males. Deletions can vary in size from the whole short arm of chromosome 18 to microdeletions. They may be terminal deletion which occur *de novo* in approximately two-thirds of cases, or be the result of an unbalanced translocation with the loss of 18p due to mal-segregation of parental chromosome rearrangement (balanced translocation or inversion), or ring chromosome 18 [3].

## 2. Case Report

**Figure 1:**
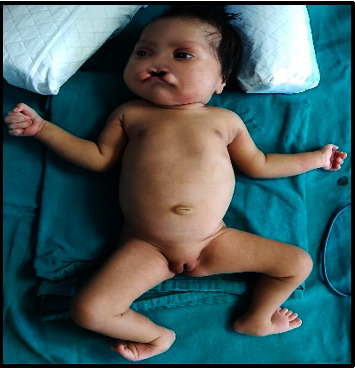
Neonate with 18p 11.1 microdeletion syndrome.

**Figure 2:**
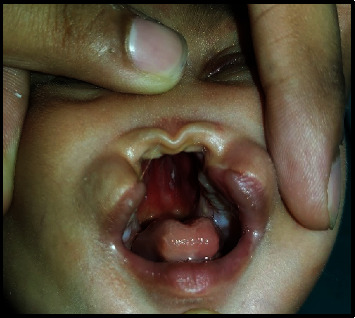
Complete cleft upper lip with complete cleft hard and soft palate.

**Figure 3:**
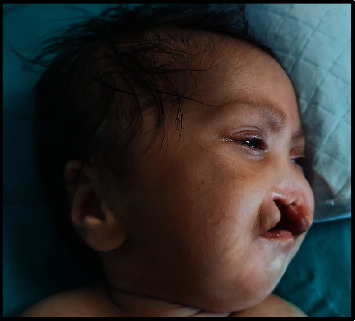
Low set ears.

**Figure 4:**
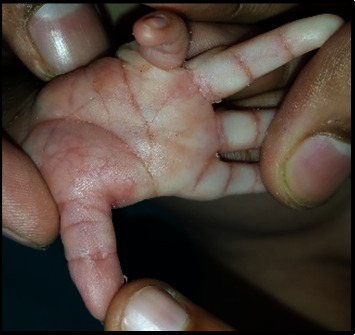
Simian crease in hand.

**Figure 5:**
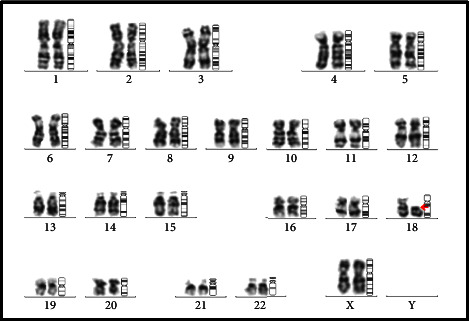
Karyogram showing 46, XX, deletion of (18) (p11.1) karyotype, suggestive of a possible presence of a terminal deletion of chromosome 18 with the breakpoint located at the 18p11.1 region.

**Figure 6:**
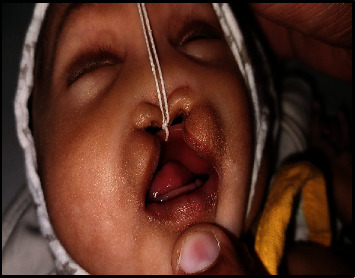
Obturator was placed to separate nasal and oral cavity.

A full-term female neonate was delivered by lower segment cesarean section (LSCS) due to previous LSCS. Parents were healthy and nonconsanguineous, and the 31-year-old mother had 2 previous live normal male offsprings without any abnormalities. The present baby showed a normal adaptation to extrauterine life, had a low birth weight (LBW, 2150 gm) and was admitted to the neonatal intensive care unit (NICU) for observational care due to multiple gross congenital anomalies and to rule out other possible malformations. Antenatal ultrasonography (USG), performed at 33 weeks of gestation, detected mid-face hypoplasia, central cleft lip and palate, and mid-face fusion defect. Anthropometry at birth revealed that the baby was small for gestational age, with a weight less than 3^rd^ centile and a ponderal index of 2.35 gm/cm^3^, microcephaly (head circumference 31 cm, 3^rd^ centile), and normal length (47 cm, between the 10^th^ and 50^th^ centile), as measured by Fenton newborn growth chart for girls. [4] Clinical examination disclosed abnormal facial features including flat rounded face, absent nose, downturned corners of the mouth (Figure 1), cleft upper lip and palate (hard and soft palate) (Figure 2), microcephaly, bilateral iris coloboma, and low set ears (Figure 3). Other abnormalities were observed including central hypotonia, redundant skin over the nape of the neck, simian crease in the hand (Figure 4), clitoromegaly, bilateral congenital Talipes Equino Varus (CTEV) with rocker bottom feet, 4th finger overlapping to the 3rd and 5th ones. Plain chest skiagram and ultrasonography (USG) of the abdomen were normal. 2D-ECHO showed a 5 mm sized Ostium Secondum—Atrial Septal Defect (OS-ASD). Head USG and brain magnetic resonance imaging (MRI) showed normal findings. Whole body Xray including spine lateral and anteroposterior view resulted in normal, and bone age was reported as more than 36 weeks of gestational maturity. Ophthalmological evaluation revealed bilateral iris coloboma. The routine blood investigation and thyroid profile were normal. The standard karyotype analysis was suggestive of chromosome 18p 11.1 deletion, than confirmed by Fluorescence In Situ Hybridization (FISH) (Figure 5). CGH for precise genomic characterization was not carried out due to financial constraints of the patient. Oto acoustic emission (OAE) identified no abnormalities in both ears. Concerned specialty opinions and advice were taken to review ongoing treatment and plan an upcoming follow-up. The baby was initially kept on gavage feeding with expressed breast milk, then shifted to paladai feeding. Kangaroo mother care (KMC) was provided, in light of the low birth weight, which contributed in establishing a good bond with the daughter. She was immunized with birth vaccines (BCG and OPV zero dose). Parents were counselled about the condition of their baby, who was discharged at day 14 of life on paladai feeding with expressed breast milk, a weight gain of 90 gm (2240 gm), and calcium and multivitamin (including vitamin D) oral supplementation. Obturator was made and placed on follow-up dental visit which helped in feeding the baby effectively, resulting in adequate weight gain (Figure 6). First surgical correction for cleft lip and soft palate is planned to be performed at around 5 months of age, while the second surgical correction for hard palate and gum pad at around 1.5 years of age, as per pediatric surgeon and plastic surgeon advices [5]. The parents were advised to undergo genetic testing, but they refused. The potential future developmental concerns of the baby were duly informed to the parents and regular close follow-up was explained.

## 3. Discussion

18p deletion syndrome was first reported in 1963 by the French geneticist Jean de Grouchy, and hence, it is also known as de Grouchy syndrome. Clinical features vary considerably within patients. The 18p deletion syndrome survival is variable, ranging from a few months to several decades [6]. The majority of patients (80%) show minor malformations and mild intellectual disability [7]. According to literature, major abnormalities associated with 18p deletion syndrome are hypotonia and microcephaly (29%), epicanthal fold (40%), hypertelorism (41%), micrognathia (25%), CTEV (13%), cardiac defect (10%) [2]. In our case, the features consistent with 18p deletion included microcephaly, hypotonia, CTEV, OS-ASD, simian crease of the hand, turning corners of the mouth, rounded face, and cleft lip and palate. Additional findings of our proband were as follows: absent nose, bilateral iris coloboma, low set ears, skin redundancy over the nape of the neck, rocker bottom right foot, 4th finger overlapping with the 3rd and 5th ones, and clitoromegaly. In our female newborn, most of the phenotypic characteristics (low birth weight, skin redundancy, ASD, microcephaly, cleft lip and palate, simian crease, CTEV, rocker-bottom foot, and iris coloboma) overlapped with those of trisomy 18 (Edwards' Syndrome), which must be included in the differential diagnosis. However, the genotype of the proband was diagnosed as chromosome 18p 11.1 microdeletion. Newer modalities are available to identify such deletion, i.e., array comparative genomic hybridization (aCGH) testing [3]. Hasi-Zogaj et al. reviewed 106 18p deletion patients, and found that seizures were not common [8]. Other reported features seen in later life were mild-to-moderate growth deficiency due to growth hormone deficiency, ptosis, alopecia, dental caries, intellectual disability, emotional liability, language impairment, dystonia, schizophrenia, immunological disorders such as IgA absence or deficiency, cataract, and strabismus [2]. In a cohort analysis of subjects with 18p deletions, Hasi-Zogaj et al. discovered that 89% had *de novo* isolated deletions [9]. However, partial deletions may also be subsequent to unbalanced translocations. In these cases the phenotype may be influenced by the accompanying trisomy, thus explaining the clinical diversity observed in the 18p syndrome [10]. Other factors that may affect this variability include the patients' varying ages, undiagnosed mosaicism, and unmasking of a recessive trait by the deletion [6].  Table 1 displays several genomic (chromosomal microdeletions) or epigenetic mutations associated with different genetic diseases or malformative syndromes.

To prevent the complications due to the abovementioned health issues, multidisciplinary care is advised for these patients, including paediatrician, dentist, orthopaedic, plastic surgeon, child psychologist, dermatologist, ophthalmologist, cardiologist, endocrinologist, pediatric neurologist and otorhinolaryngologist. Such multidisciplinary aproaches will provide prompt identification of the associated potential complications and their timely management, resulting in increased survival and quality of life for the affected subjects and their families.

## Figures and Tables

**Table 1:** Different cases depicting the (epi) genotypical profiles of various genetic diseases or malformative syndromes (contiguous gene syndromes).

	Present case	Serra et al. [11]	Piro et al. [12]	Serra et al. [13]	Serra et al. [14]
Genetic test performed	Karyotyping and FISH	Methylation sensitive—multiplex ligation-dependent probe amplification (MS-MLPA)	Array comparative genomic hybridization (a-CGH)	Array comparative genomic hybridization (a-CGH)	Array comparative genomic hybridization (aCGH)
Genotype	18p 11.1 microdeletion	Hypomethylation of KCNQ1OT1, with a normal pattern of methylation of the imprinting center (IC) 1	2q13 deletion of 1.7 Mb	19p13.3 microdeletion, of 1.27 Mb and including MAP 2 K2 gene	Terminal deletion at 11q24.1-q25
Phenotype resembling	Trisomy 18	Beckwith–Wiedemann syndrome	2q13 deletion	Cardio-facio-cutaneous syndrome (CFCS)	Jacobsen syndrome

## Data Availability

The data sharing is not applicable to this article as no new data were created or analysed in this study.
